# From the First 1000 Days to the First 100 Years: Early-Life Biological Programming, Sentinel Phenotypes, and Healthy Aging

**DOI:** 10.3390/children13101302

**Published:** 2026-09-25

**Authors:** Luca Pecoraro, Sebastiano Salaorni, Sara Mozzon, Riccardo Russo, Gabriele Renzetti, Flavia Indrio, Giorgio Piacentini, Angelo Pietrobelli

**Affiliations:** 1 Department of Experimental Medicine, Pediatric Section, University of Salento Hospital “Vito Fazzi”, 73100 Lecce, Italyflavia.indrio@unisalento.it (F.I.); 2 Pediatric Division, Department of Surgical Sciences, Dentistry, Gynecology and Pediatrics, University of Verona, 37126 Verona, Italy; giorgio.piacentini@univr.it (G.P.);; 3 Primary Care Pediatrician, Azienda ULSS 9 Scaligera, 37126 Verona, Italy; 4 Pennington Biomedical Research Center, Baton Rouge, LA 70808, USA

**Keywords:** first 1000 days, early-life programming, biological aging, healthy aging, oxidative stress, mitochondrial dysfunction, telomere length, epigenetics, chronic inflammation, life-course medicine

## Abstract

**Highlights:**

**What are the main findings?**

- The first 1000 days represent a critical period of biological plasticity during which early-life exposures may shape lifelong health trajectories through epigenetic, mitochondrial, inflammatory, oxidative, and telomere-related mechanisms.
- Allergic and atopic disease, respiratory vulnerability, renal–cardiovascular risk, and altered metabolic growth may represent illustrative pediatric “sentinel phenotypes” of altered developmental trajectories.

**What are the implications of the main findings?**

- Early-life programming should be considered a modifiable component of lifelong health rather than a deterministic predictor of adult disease.
- Prevention during pregnancy, infancy, and early childhood may contribute to healthier developmental trajectories and biological aging.

**Abstract:**

The first 1000 days of life, spanning from conception to approximately two years of age, represent a period of exceptional biological plasticity during which nutritional, metabolic, inflammatory, psychosocial, microbial, and environmental exposures may shape developmental trajectories across the life course. For this narrative review, PubMed/MEDLINE, Scopus, and Web of Science were searched from inception to June 2026, focusing primarily on human cohorts, systematic reviews, meta-analyses, guidelines, and consensus documents, with selected experimental studies included for mechanistic evidence. Within the framework of the Developmental Origins of Health and Disease (DOHaD), we examine the epigenetic, oxidative, inflammatory, mitochondrial, and telomere-related mechanisms proposed to link early-life exposures with lifelong health and biological aging. Because most human evidence in this field is observational, whereas much of the mechanistic evidence derives from experimental models, these relationships are presented as associations and biologically plausible hypotheses rather than as demonstrated causal pathways. We then consider how these processes may become clinically apparent during childhood through four illustrative “sentinel phenotypes”—allergic and atopic disease, respiratory vulnerability, renal–cardiovascular risk, and altered metabolic growth—proposed here as a conceptual framework rather than as an established classification. We also review preventive opportunities during pregnancy, infancy, and early childhood. Overall, the first 1000 days should be viewed as a critical window for developmental health, while the specific contribution of this review is to integrate early-life biological mechanisms with clinically observable pediatric sentinel phenotypes within the broader framework of biological and healthy aging.

## 1. Introduction

The first 1000 days, conventionally spanning from conception to approximately two years of age, represent a period of exceptional developmental plasticity during which environmental signals can influence biological trajectories with potentially lifelong consequences. Rapid cellular proliferation, organogenesis, tissue remodeling, and functional maturation make developing biological systems particularly responsive to environmental conditions, with distinct windows of susceptibility across organs and physiological systems [1,2]. The concept that adult disease may have developmental origins emerged from epidemiological observations linking adverse early-life conditions with increased cardiovascular risk and mortality later in life [3]. Barker and Osmond further developed this concept in 1986 [4], contributing to the Developmental Origins of Health and Disease (DOHaD) framework [5]. Developmental programming should not be interpreted simply as damage caused by adverse exposures, but rather as the capacity of the developing organism to adjust its structure and physiology in response to environmental conditions, potentially generating trade-offs between immediate adaptation and long-term health. Molecular and cellular processes implicated in aging, including oxidative stress, inflammation, mitochondrial dysfunction, epigenetic alterations, telomere dynamics, and cellular senescence, may also be influenced by early-life exposures during periods of heightened biological plasticity [1,6]. Recent evidence supports this life-course perspective. A systematic review and meta-analysis including 344,852 participants found a significant association between adverse early-life circumstances and measures of multisystem biological aging, although substantial heterogeneity was observed according to exposure type, timing, and methods used to assess biological aging [7]. These findings support an association between early-life conditions and later biological aging but do not establish that early-life characteristics determine individual aging trajectories or lifespan. The first 1000 days may therefore represent an early biological window during which trajectories relevant to later resilience and vulnerability begin to emerge [7,8,9]. Although the DOHaD framework and the long-term relevance of the first 1000 days are well established, the specific integration of early-life biological mechanisms with clinically observable pediatric sentinel phenotypes within the broader framework of biological and healthy aging remains less clearly synthesized. The aim of this narrative review was therefore to integrate current evidence on early-life exposures and biological mechanisms with four illustrative pediatric sentinel phenotypes as clinically observable manifestations of altered developmental trajectories relevant to later resilience, vulnerability, and healthspan. By bringing these dimensions together, this review proposes a life-course framework linking early-life biology with pediatric clinical phenotypes and biological aging, while explicitly recognizing that these relationships are not deterministic and that direct longitudinal evidence across the human lifespan remains limited.

## 2. Literature Search Methodology

A non-systematic narrative literature search was performed in PubMed/MEDLINE, Scopus, and Web of Science from database inception to June 2026. Search terms included “first 1000 days”, “developmental origins of health and disease”, “early-life programming”, “epigenetics”, “oxidative stress”, “inflammation”, “mitochondrial dysfunction”, “telomere length”, “cellular senescence”, and “biological aging”, combined with terms related to relevant biological mechanisms and clinical outcomes using Boolean operators (AND/OR). Search terms were adapted to the indexing and search syntax of each database. No lower publication-date limit was applied. Studies addressing early-life exposures or developmental processes and their relevant biological, developmental, or clinical outcomes were considered. The evidence base focused primarily on human cohort studies, systematic reviews, meta-analyses, consensus documents, and clinical or public-health guidelines, while selected experimental and animal studies were included to provide mechanistic evidence. Studies unrelated to early-life programming, the biological mechanisms examined, or the clinical outcomes of interest were excluded. Literature identification and selection were conducted by S.S., S.M., and R.R., who performed three separate searches, with the overall process supervised by the remaining authors. Relevant articles were assessed through title/abstract and full-text review, and additional studies were identified from the reference lists of key publications. Given the descriptive nature of this narrative review, a formal risk-of-bias assessment was not performed. When evidence conflicted, greater weight was given to systematic reviews, meta-analyses, prospective cohort studies, and established clinical guidelines. ChatGPT (GPT-5.6 Luna, OpenAI) assisted with the preparation and refinement of Figure 1. The authors reviewed and edited the generated content and take full responsibility for the final version.

## 3. The Biology of the First 1000 Days

During fetal life, the placenta plays a central role in developmental programming by integrating maternal nutritional, metabolic, endocrine, and inflammatory signals and regulating their transmission to the fetus. Maternal obesity and gestational weight gain have been associated with differences in placental DNA methylation, including at loci involved in metabolic regulation [10,11]. Similarly, gestational diabetes has been associated with altered DNA methylation patterns in the placenta and cord blood [12]. These findings suggest that epigenetic mechanisms may act as important modulators of fetal metabolic programming and could potentially contribute to later susceptibility to adverse metabolic outcomes, although the long-term consequences of these epigenetic differences remain incompletely established. Environmental pollutants and maternal infection provide further examples of how prenatal exposures may influence developing biological systems. Prenatal exposure to particulate matter has been associated with altered mitochondrial DNA (mtDNA) content in newborns [13], while recent whole-mitochondrial-genome analyses have identified associations between gestational nitrogen dioxide (NO_2_) exposure and cord-blood mtDNA heteroplasmy during specific gestational windows [14]. Maternal immune activation may also produce persistent biological effects. In a murine model, maternal infection induced IL-6-dependent epigenetic changes in fetal intestinal epithelial stem cells, resulting in offspring with enhanced antimicrobial protection but increased susceptibility to intestinal inflammation [15]. These findings illustrate how the same early adaptation may be beneficial in one environmental context while increasing vulnerability in another. Birth does not mark the end of developmental programming but rather represents a profound transition in its mediators. The neonate shifts from a maternally buffered intrauterine environment to direct interaction with oxygen, nutrition, microorganisms, pathogens, sensory experiences, and psychosocial signals, while major physiological systems remain highly plastic. The brain is among the organs undergoing particularly rapid postnatal development, with total brain volume approximately doubling during the first year of life and continuing to increase during the second year [16]. While neural architecture is actively constructed and reorganized, nutrition, sensory stimulation, caregiver interactions, and sleep provide additional environmental inputs that may influence developmental trajectories. Recent evidence further supports the concept that neurodevelopment, gut maturation, and sleep regulation should not be considered independent processes during the first 1000 days. A recent review described a bidirectional brain–gut–sleep network in which nutritional, microbial, metabolic, and neuroendocrine signals interact during early development, potentially influencing cognitive, behavioral, and sleep trajectories [17]. In parallel, the immune system must develop effective antimicrobial responses while establishing tolerance to food antigens and commensal microorganisms. The gut microbiome plays an important role in this process and is influenced by multiple early-life factors, including mode of delivery, breastfeeding, antibiotic exposure, and complementary feeding [18,19,20]. These effects are dynamic: feeding practices, for example, can substantially modify initial differences in microbiota associated with mode of delivery [19]. The establishment of the gut microbiome, therefore, represents a continuously evolving process in which maternal, nutritional, microbial, and environmental factors interact throughout infancy. Breast milk represents a particularly complex source of postnatal biological information. In addition to nutrients, immunoglobulins, cytokines, and growth factors, human milk contains human milk oligosaccharides (HMOs), which are largely indigestible by the infant but selectively interact with intestinal microorganisms. Human studies have linked HMO profiles with infant microbiota composition [21,22], while maternal diet itself may influence milk oligosaccharide composition [23]. Accordingly, the biological dialogue between mother and child continues well beyond childbirth, although through mechanisms that differ from those operating during fetal life. Around six months of age, complementary feeding constitutes another major developmental transition. The introduction of new nutrients and food antigens is accompanied by changes in nutrient availability and microbial substrates, with potential consequences for metabolic signaling and microbiota development. Recent human studies have linked complementary feeding with microbial and metabolomic changes during infancy [24]. This transition represents an additional opportunity for environmental factors to interact with highly plastic biological systems. The first two postnatal years can therefore be viewed as a critical window during which programming progressively shifts from predominantly maternally mediated signals toward increasingly direct environmental influences. Brain maturation, immune education, microbial assembly, epithelial-barrier development, nutritional transitions, and metabolic adaptation are deeply interconnected processes. Within a conceptual life-course framework, these processes may contribute to physiological reserve and the capacity to respond appropriately to future environmental and metabolic challenges. This capacity can be conceptualized as biological resilience: the ability of developing biological systems to maintain or recover functional homeostasis in response to environmental perturbations. Conversely, adverse or poorly matched exposures during critical developmental windows may increase biological vulnerability. Such resilience should not be regarded as the established consequence of any single early-life exposure or pathway, but rather as a conceptual construct that may emerge from interactions among genetic susceptibility, developmental timing, nutrition, metabolism, immune maturation, microbial ecology, and the broader social and physical environment.

## 4. Oxidative Stress, Chronic Inflammation, and Mitochondrial Dysfunction: Linking Early-Life Programming to Aging

Oxidative stress and chronic low-grade inflammation are increasingly recognized as interconnected processes that contribute to the loss of cellular homeostasis and the development of age-related disease. Mitochondria occupy a central position within this network, linking energy metabolism and redox regulation with innate immune signaling, cellular stress responses, and epigenetic regulation. Understanding this interplay may provide an important mechanistic bridge between developmental programming during the first 1000 days and biological vulnerability later in life. Beyond ATP production, mitochondria regulate intermediary metabolism, calcium homeostasis, apoptosis, redox signaling, and innate immunity. Furthermore, mitochondrial metabolites can influence epigenetic enzymes and gene regulation [25,26,27,28,29]. Reactive oxygen species (ROS) should not be considered merely harmful metabolic by-products. At physiological concentrations, ROS participate in cellular signaling, including the regulation of proliferation, differentiation, and adaptive responses [27,28]. However, when redox homeostasis is disrupted, excessive or sustained ROS production may contribute to molecular damage and dysregulated stress signaling. Persistent oxidative stress can damage proteins, lipids, and nuclear and mitochondrial DNA, while mitochondrial injury may further increase ROS generation, creating a self-amplifying cycle of mitochondrial dysfunction [26,30]. Mitochondrial quality-control mechanisms, including fusion, fission, and selective elimination of damaged mitochondria through mitophagy, enable cells to maintain mitochondrial integrity under stress. When these mechanisms become insufficient or dysregulated, damaged mitochondria may initiate inflammatory signaling. The accumulation of dysfunctional, ROS-producing mitochondria can promote activation of the NLRP3 inflammasome [30]. In addition, mitochondrial damage can result in the release or inappropriate exposure of mitochondrial DNA (mtDNA), which functions as a damage-associated molecular pattern (DAMP). Cytosolic mtDNA can activate the cyclic GMP-AMP synthase–stimulator of interferon genes (cGAS–STING) pathway, thereby linking mitochondrial damage to innate immune signaling [25]. Recent evidence further supports the concept that mitochondrial integrity and mitophagy act as important checkpoints that restrain mtDNA-dependent inflammatory signaling during aging, although the relationship between mitophagy and aging is more complex than a simple age-related decline in mitophagic activity [31,32]. Mitochondrial dysfunction and altered redox homeostasis may therefore contribute to innate immune activation and inflammation, establishing a potentially self-reinforcing cycle of mitochondrial and metabolic stress. This interaction becomes particularly relevant during aging, when the capacity of biological systems to maintain homeostasis and recover from stress may progressively decline. Mitochondrial dysfunction, altered mitochondrial quality control, cellular senescence, and chronic low-grade inflammation are closely interconnected features of this process. Recent reviews have emphasized mitochondria as central hubs linking oxidative stress, inflammation, mitochondrial DNA damage, altered mitochondrial dynamics, mitophagy, and aging, reinforcing their potential role as a mechanistic interface between cellular dysfunction and age-related disease [33]. Experimental evidence increasingly supports a causal contribution of mitochondrial innate immune signaling to aging phenotypes. In mtDNA-mutator mice, enhanced type I interferon responses aggravated metabolic dysfunction, inflammation, and premature aging phenotypes [26]. Conversely, in experimental models of ataxia telangiectasia, NAD+ supplementation improved mitophagy and attenuated STING-associated senescence and inflammatory signaling [34]. Gulen et al. demonstrated that cGAS–STING signaling contributes to age-associated inflammation and functional decline: inhibition of STING reduced inflammatory responses in aged mice and attenuated neurodegenerative phenotypes [35]. These findings have been reinforced by subsequent studies showing that cytosolic mtDNA accumulation and cGAS–STING activation increase during aging, whereas effective mitochondrial quality control can limit mtDNA-dependent inflammatory signaling [31]. More broadly, recent work continues to position cGAS–STING as an important regulator of age-associated inflammation, cellular senescence, and tissue dysfunction across experimental models [33,36]. These findings offer a plausible biological continuum connecting developmental plasticity with aging, but they should not be interpreted as evidence that mitochondrial changes established during infancy directly determine human lifespan. Accordingly, the proposed sequence linking prenatal or early-life exposures, molecular alterations, childhood phenotypes, adult disease, and biological aging should be regarded as a conceptual model rather than an established longitudinal pathway, as its individual links are supported by distinct lines of evidence and have rarely been demonstrated within the same human populations across the life course. Human and experimental studies show that prenatal and early-life exposures are associated with differences in epigenetic, mitochondrial, metabolic, and inflammatory phenotypes [10,11,12,13,14,15,37], whereas experimental models demonstrate that specific mitochondrial quality-control and innate immune pathways can causally contribute to cellular and organismal aging phenotypes [26,29,31,35,38,39,40]. Importantly, emerging human evidence suggests that adverse childhood experiences may be associated with mitochondrial dysfunction detectable decades later. In a cohort of older adults, a greater burden of adverse childhood experiences was associated with lower skeletal muscle ATP production, suggesting that early-life stress may leave persistent signatures in mitochondrial function, although these findings cannot establish causality or be directly extrapolated to the first 1000 days [41]. Directly connecting these observations across a century of human life remains an important research challenge. Nevertheless, taken together, these distinct lines of evidence provide biological plausibility for the hypothesis that early nutritional, metabolic, microbial, inflammatory, and environmental conditions may influence the capacity to maintain redox balance, mitochondrial quality, and appropriate immune regulation when challenged later in life [10,11,12,13,14,15,25,26,27,29,30,31,34,35,36,37,38,39,40]. From this perspective, the biological foundations of healthy longevity may begin long before the clinical manifestations of age-related disease. These observations underscore the importance of healthy development during pregnancy and the first two years of life, while the extent to which modifying specific early-life exposures can produce more resilient biological trajectories across the life course remains to be established [10,11,12,13,14,15,16,17,18,19,20,21,22,23,24,37]. Importantly, mitochondrial function and epigenetic regulation are not independent processes: mitochondrial metabolites can modulate the activity of chromatin-modifying enzymes, while epigenetic mechanisms can, in turn, regulate mitochondrial biogenesis and stress responses [42]. This bidirectional interaction represents a plausible molecular interface through which early-life exposures may influence long-term biological trajectories.

## 5. Epigenetic Mechanisms of Early-Life Programming

Epigenetic regulation represents one of the main mechanisms through which early-life conditions may influence gene activity beyond the period of the original exposure [43]. The principal mechanisms include DNA methylation, post-translational histone modifications, chromatin remodeling, and regulation by non-coding RNAs [44,45]. These processes are particularly dynamic during gametogenesis, embryogenesis, placentation, and early postnatal development, when cells acquire their identity and organs undergo rapid differentiation. Nutritional imbalance, maternal metabolic disease, psychosocial stress, and environmental exposures may therefore interact with developmental programs during periods of marked biological plasticity. DNA methylation is the most widely studied epigenetic mechanism in human cohorts, partly because it can be measured in placenta, cord blood, and peripheral blood. Methylation at CpG sites may influence transcription-factor binding and gene expression, although its functional consequences depend on genomic location, cell type, tissue, and developmental stage. Histone acetylation and deacetylation influence chromatin structure and DNA accessibility, while microRNAs and long non-coding RNAs regulate transcription and messenger RNA stability [44]. Recent evidence further emphasizes that epigenetic alterations in early life are highly tissue- and exposure-specific, highlighting the importance of biological context when interpreting associations between early-life exposures and DNA methylation patterns [45]. Natural experiments provide some of the strongest human evidence that prenatal conditions may be associated with long-lasting molecular signatures. Individuals exposed around conception during the Dutch Hunger Winter showed differences in methylation of the imprinted IGF2 locus almost six decades later [37]. This finding indicates that a relatively restricted period of prenatal undernutrition may be associated with persistent epigenetic differences. However, it does not demonstrate that these differences were directly responsible for the cardiometabolic disorders observed later in life. Evidence from less extreme nutritional exposures is more variable. Human studies reported associations between maternal prenatal diet and infant DNA methylation, but the evidence was of low certainty, with substantial variation in dietary assessment, biological tissue, analytical methods, control for confounding, and risk of bias [46]. These findings are consistent with an association between prenatal nutrition and epigenetic variation, although the long-term clinical consequences of many of these changes remain uncertain. Several pathways may link maternal nutrition to fetal growth and glucocorticoid signaling. An unbalanced maternal diet has been associated with differences in offspring methylation of genes involved in glucocorticoid action and growth, including those encoding 11β-hydroxysteroid dehydrogenase type 2, the glucocorticoid receptor, and IGF2 [47]. Placental glucocorticoid metabolism normally limits fetal exposure to maternal cortisol. Alterations in this protective mechanism may influence hippocampal maturation and the hypothalamic–pituitary–adrenal axis, potentially affecting stress responsivity, cognition, blood pressure regulation, and metabolism. Evidence for this pathway derives largely from experimental animal models and has not been demonstrated longitudinally in humans [48,49]. These associations are unlikely to reflect changes in a single gene; rather, endocrine, inflammatory, nutritional, and oxidative signals may interact across multiple tissues during development, as experimental evidence suggests that HPA axis activation itself induces cellular oxidative stress [50]. Experimental models are particularly useful for disentangling individual mechanisms. In human pregnancy, nutritional, placental, genetic, and social factors often occur together and are difficult to study in isolation. Animal models allow researchers to follow a defined exposure through specific tissues and molecular pathways. In a rat model of intrauterine growth restriction, progressive epigenetic silencing of Pdx1 was associated with impaired pancreatic development and increased susceptibility to diabetes; within this experimental model, the sequence is consistent with a causal mechanism, but it cannot be extrapolated directly to humans [51]. In other experimental models, maternal diet modified adipose-tissue microRNA expression, with potential consequences for adipogenesis and insulin signaling [52]. In a rat model of early-life exposure to an endocrine-disrupting chemical, reprogramming of hepatic histone marks accelerated the acquisition of an adult epigenomic signature; however, the associated metabolic dysfunction became apparent only after a later dietary challenge in adulthood [53]. These models provide important mechanistic insights, although their findings cannot be directly extrapolated to human populations because of differences in developmental timing, exposure intensity, tissue specificity, and disease phenotype. The human phenotype is particularly heterogeneous among infants born small for gestational age (SGA). Reduced birth size may reflect constitutional characteristics or different combinations of placental insufficiency, maternal disease, malnutrition, genetic factors, and imprinting disorders. Therefore, the biological consequences of SGA cannot be attributed to birth size alone. The international SGA consensus identifies accelerated postnatal weight gain as an important risk factor consistently associated with later cardiometabolic risk [54]. From a developmental perspective, this may reflect a mismatch between prenatal adaptation and the postnatal environment, whereby rapid nutritional recovery after a period of fetal constraint may increase metabolic vulnerability. This concept is consistent with the broader DOHaD framework, in which the health consequences of an early-life adaptation depend not only on the original exposure but also on subsequent environmental conditions [53]. An important extension of this concept is the possibility that epigenetic mechanisms may contribute not only to disease susceptibility but also to biological aging trajectories. DNA methylation changes systematically with chronological age, and patterns of age-associated CpG methylation have been used to develop so-called epigenetic clocks, which estimate biological age from molecular signatures. A systematic review of pediatric studies identified 68 eligible studies examining epigenetic clocks in children and childhood exposures. Although findings were mixed, most studies reported significant associations between early-life exposures and epigenetic age, or between epigenetic age and health outcomes [55]. These findings suggest that early developmental conditions may leave molecular signatures detectable not only as disease-related epigenetic changes but also as differences in biological age. However, epigenetic clocks should currently be interpreted with caution. They are biomarkers derived from statistical associations between DNA methylation patterns and chronological age and do not necessarily directly measure the biological mechanisms underlying aging. Recent methodological work has emphasized that different epigenetic clocks capture partially distinct biological processes and that their interpretation in perinatal and pediatric populations requires particular caution because DNA methylation is highly dynamic during development [56]. Nevertheless, their application to pediatric and longitudinal cohorts provides a potentially valuable framework for investigating whether exposures during the first 1000 days are associated with subsequent differences in biological aging trajectories. Taken together, current evidence is compatible with a conceptual model in which epigenetic regulation may act as a dynamic interface between the early-life environment and long-term biological function. This model integrates experimental and observational evidence and should therefore be regarded as biologically plausible rather than as demonstrated in humans. Rather than a fixed molecular imprint, epigenetic programming should be viewed as a potentially reversible, context-dependent process in which nutritional, metabolic, hormonal, inflammatory, and environmental signals interact with developmental biology. This perspective provides a mechanistic hypothesis linking the first 1000 days to the subsequent life course, while highlighting the need for longitudinal human studies to determine whether early-life epigenetic signatures predict clinically meaningful outcomes across adulthood and aging.

## 6. Telomere Biology and Early-Life Determinants of Cellular Aging

Telomere biology extends the concept of early-life programming from gene regulation and cellular metabolism to the maintenance of chromosome integrity and replicative capacity. Telomeres are repetitive DNA–protein structures that protect chromosome ends from degradation and inappropriate DNA-damage responses. Because conventional DNA replication cannot fully replicate the ends of linear chromosomes, telomeres progressively shorten with successive cell divisions in most somatic cells [57]. When telomeres become critically short or dysfunctional, persistent DNA-damage signaling may induce cellular senescence or apoptosis. Telomerase can partially restore telomeric DNA in germ cells, stem-cell compartments, and some activated immune cells, but its activity is limited in most somatic tissues. Importantly, telomere length is not determined solely by cumulative cellular replication after birth. A substantial proportion of inter-individual variation in telomere length is already present at or around birth and has been attributed to both genetic inheritance and prenatal biological conditions. A large meta-analysis including 19,713 individuals estimated telomere-length heritability at approximately 70%, with evidence of both maternal inheritance and an association with paternal age at birth, supporting a substantial genetic and parental contribution to telomere length [58]. Studies of childhood telomere biology have also reported substantial inter-individual differences already present among newborns, suggesting that intrauterine development may be an important period in determining telomere length [59]. Thus, although telomere length is established early in life, telomere attrition continues throughout the life course. A systematic review and meta-analysis of 414 study samples, including more than 743,000 individuals, reported an overall inverse association between telomere length and chronological age across the human lifespan, together with substantial heterogeneity in telomere trajectories and measurement-related effects [60]. The same oxidative and inflammatory environment discussed in previous sections may influence telomere maintenance. In experimental systems, reactive oxygen species can damage telomeric DNA, which is particularly susceptible to oxidative injury, potentially accelerating telomere attrition [57]. Maternal or infant undernutrition may influence antioxidant defenses and mitochondrial function, whereas maternal metabolic disease, nutrient excess, and rapid postnatal weight gain may increase inflammatory and oxidative stress. Early nutrition has therefore been hypothesized to influence telomere dynamics indirectly through oxidative and inflammatory pathways; however, this remains a mechanistic hypothesis rather than a pathway demonstrated in humans. Evidence linking nutrition directly to telomere length is considerably less consistent than that linking early-life nutrition to epigenetic regulation. A systematic review specifically examining maternal diet and offspring telomere length identified only seven studies and found heterogeneous associations, providing limited and suggestive rather than conclusive evidence [61]. Similarly, a systematic review and meta-analysis of diet and telomere length found insufficient evidence to establish a consistent effect of diet on telomere length, further supporting caution in interpreting nutrition as a direct determinant of telomere maintenance [62]. Intervention studies during pregnancy have focused primarily on birth weight, growth, adiposity, blood pressure, and cardiovascular outcomes [63], with telomere length rarely included as an outcome. Nutrition should therefore be considered an upstream influence on the biological environment in which telomeres are maintained, rather than an established intervention for preventing telomere shortening. Telomere length also changes dynamically during early growth and varies substantially between individuals. Longitudinal studies suggest that telomere trajectories are not necessarily linear and may vary with inherited telomere length, baseline values, growth rate, immune cell composition, and environmental exposures [64]. Prenatal psychological stress has received particular attention as a potential early-life determinant of telomere biology. A systematic review and meta-analysis including eight observational studies found an inverse association between maternal psychological stress during pregnancy and newborn telomere length. The quantitative synthesis of four studies showed a small reduction in newborn telomere length with increasing maternal stress, although the pooled estimate was sensitive to individual datasets and the overall evidence remained heterogeneous [65]. These findings support a possible association between maternal stress and fetal telomere biology but do not establish that prenatal stress directly causes accelerated telomere shortening or premature biological aging. Researchers have also investigated birth size and postnatal growth as potential determinants, but the available evidence remains inconsistent. A systematic review and meta-analysis of early-life adiposity found no convincing longitudinal association between birth weight and later telomere length, with only limited evidence regarding differences between children born small for gestational age and those born appropriate for gestational age [66]. These findings reinforce the need to distinguish biological plausibility from established causal relationships and suggest that SGA and postnatal catch-up growth should not be interpreted as independent determinants or as established causes of telomere shortening. Telomere length is thought to reflect several aspects of cellular history, including replication, oxidative injury, inflammation, and hematopoietic turnover. It is not, however, a precise measure of future aging. Estimates depend on tissue selection, leukocyte composition, assay technique, chromosome-end specificity, and age at sampling. Interpreting telomere length as a biomarker of biological aging therefore requires caution, as an umbrella review found strong or moderate evidence for associations with only a limited number of health outcomes and substantially weaker evidence for many others [67]. Early adversity has been hypothesized to be associated with reduced cellular reserve or greater vulnerability to subsequent stress, but nutrition, physical activity, disease, psychosocial conditions, and environmental exposures continue to influence telomere dynamics throughout the life course. Telomere biology should therefore be viewed as a dynamic lifelong process in which early developmental conditions may influence initial telomere reserve without irrevocably determining subsequent aging. Overall, the association between early-life conditions and telomere biology is supported largely by cross-sectional and observational human data, complemented by experimental evidence on oxidative telomere damage. The resulting model is therefore biologically plausible, but a causal contribution of early-life exposures to telomere-related biological aging in humans remains to be demonstrated.

## 7. Pediatric Implications: Early-Life “Sentinel Phenotypes”

The biological processes described above are not restricted to molecular or cellular phenotypes. Their clinical consequences may become apparent during childhood, sometimes as early manifestations of disease trajectories that extend across the life course. From this perspective, pediatric diseases may be viewed as “sentinel phenotypes”: clinically recognizable manifestations that may provide early clues to altered biological trajectories rather than deterministic predictors of adult disease. In this review, we propose a conceptual framework in which selected pediatric conditions serve as illustrative “sentinel phenotypes” that link early-life biological programming to clinically observable trajectories. The four phenotypes were selected by the authors based on three complementary considerations: (I) their clinical recognizability during childhood; (II) the availability of evidence linking their development to exposures or biological processes operating during the first 1000 days; and (III) their ability to represent distinct, biologically interconnected physiological domains. The relevance of the first 1000 days as a period of developmental programming is increasingly recognized across physiological systems, including renal development [68]. Accordingly, the selected examples encompass immune–epithelial, respiratory, renal–cardiovascular, and metabolic development. This selection is not intended to imply that these conditions are uniquely or disproportionately influenced by early-life programming, nor to provide an exhaustive classification of pediatric disease. Rather, these phenotypes were chosen because, taken together, they provide a clinically accessible framework for illustrating how developmental programming may become visible across different organ systems and how early biological vulnerability may manifest through distinct clinical trajectories. Other pediatric conditions may also fit this conceptual framework and could be considered in future applications of the model. Importantly, these phenotypes should not be interpreted as deterministic predictors of adult disease. Rather, they may provide early clinical markers of altered biological trajectories, identifying periods during which biological resilience may still be influenced. In this sense, the pediatric period represents not only the beginning of a potential disease trajectory but also a potential window for prevention and modification of lifelong health [1,7,8,9].

### 7.1. Allergic and Atopic Disease: From Early Immune Dysregulation to the Allergic March

Atopic dermatitis (AD) represents an important early manifestation of immune dysregulation and impaired skin barrier function. Alteration of the skin barrier facilitates the transcutaneous penetration of allergens and microorganisms, thereby contributing to sensitization and the subsequent development of food and respiratory allergies. Moreover, in children with AD, a greater oxidative stress burden, as indicated by elevated urinary levels of 8-OHdG, has been associated with an increased risk of developing allergic asthma [69]. The clinical relevance of AD may therefore extend beyond the skin, as its early appearance may identify children with a broader susceptibility to subsequent allergic disease. The concept of the allergic march provides a clinically recognizable example of how early immune and epithelial disturbances may evolve throughout childhood. Food allergy and AD frequently precede respiratory allergic disease, including asthma and allergic rhinitis, although this sequence is neither universal nor deterministic. Rather than representing independent diseases, these conditions may reflect partially overlapping developmental trajectories involving epithelial-barrier dysfunction, type 2 immune responses, microbiome–immune interactions, and genetic susceptibility. This interpretation is consistent with the broader concept of developmental plasticity described above, in which early environmental signals may influence the maturation of multiple interconnected physiological systems. The implications of AD, however, may extend beyond allergic disease. Affected children have been reported to have an increased risk of neurodevelopmental and psychiatric conditions, including attention-deficit/hyperactivity disorder (ADHD), autism spectrum disorders, depression, anxiety, and behavioral disorders [70]. Although the mechanisms underlying these associations have not yet been fully elucidated, a potential role of systemic inflammation and its interaction with the central nervous system has been hypothesized. Consistent with this hypothesis, the presence of AD during the first years of life has also been associated with alterations in motor development, suggesting a possible involvement of neuroinflammatory mechanisms [71]. Taken together, early allergic and atopic manifestations may therefore be considered sentinel phenotypes of altered immune and epithelial development. Their presence does not imply an inevitable progression toward asthma or other chronic diseases, but may identify a period in which immune maturation, barrier function, and environmental exposures remain particularly modifiable.

### 7.2. Respiratory Health: From Early-Life Wheezing to Lifelong Lung Function

Respiratory health provides another clinically relevant example of developmental programming. Respiratory syncytial virus (RSV) is one of the leading causes of lower respiratory tract infections during the first years of life. Although RSV infection is extremely common during epidemic periods, its clinical presentation is heterogeneous, and only a proportion of children develop severe bronchiolitis. Importantly, early respiratory phenotypes may reflect differences in lung development that precede the onset of clinically recognizable asthma. A prospective birth cohort demonstrated that reduced lung function measured shortly after birth was associated with an increased risk of asthma at 10 years of age, supporting the concept that respiratory vulnerability may be detectable before the clinical expression of chronic airway disease [72]. More recent birth-cohort evidence further suggests that prenatal and early childhood periods may represent important windows during which environmental exposures can influence asthma and chronic obstructive lung disease phenotypes across the life course [73]. These findings are particularly relevant because lung development and maturation extend throughout childhood. Failure to achieve adequate maximal lung function may be associated with respiratory vulnerability later in life, even in the absence of overt chronic respiratory disease during early childhood [72,73]. Thus, wheezing, recurrent lower respiratory tract disease, and reduced lung function may represent different clinical expressions along a continuum of respiratory developmental susceptibility. Early respiratory infections may also be associated with subsequent respiratory outcomes, although the strength and direction of these associations vary according to the infection, age at exposure, prematurity, host susceptibility, and environmental context. The clinical significance of an early respiratory phenotype, therefore, lies less in predicting a specific adult diagnosis than in identifying children whose respiratory trajectory may warrant closer longitudinal observation.

### 7.3. Renal Development and Cardiovascular–Kidney Risk

The kidney provides an illustrative example of how developmental programming may contribute to a long-term disease trajectory. Human nephrogenesis is largely completed by term birth, and final nephron endowment varies substantially between individuals. Consequently, conditions affecting fetal growth and kidney development may have effects that extend beyond the neonatal period. The first 1000 days have been identified as a critical period for renal development, during which maternal illness, malnutrition, environmental exposures, infection, and other stressors may influence renal programming [68,74]. Reduced nephron endowment has been proposed as one mechanism linking adverse developmental conditions with later hypertension and chronic kidney disease. In this model, early developmental constraints may reduce renal functional reserve, potentially increasing susceptibility to subsequent hemodynamic and metabolic stressors throughout the life course. This concept is particularly relevant in children born preterm or small for gestational age, although birth size alone should not be considered a direct surrogate for nephron number or future kidney disease. The renal trajectory also illustrates the importance of interactions between developmental programming and subsequent exposures. An altered early-life renal phenotype does not determine the development of hypertension or chronic kidney disease; rather, it may modify susceptibility to later environmental, nutritional, metabolic, and hemodynamic challenges. This distinction is central to the DOHaD framework and parallels the concept discussed throughout this review that developmental programming creates a context of altered vulnerability rather than an irreversible biological destiny. Renal health, therefore, represents an especially informative sentinel trajectory, as developmental changes may remain clinically silent for years before becoming apparent as hypertension, reduced renal function, or chronic kidney disease [68,74].

### 7.4. Early Growth and Adiposity: From Developmental Plasticity to Cardiometabolic Risk

Altered growth trajectories provide another well-recognized example of developmental programming. Maternal metabolic disease, excessive or insufficient fetal growth, and rapid postnatal weight gain have all been associated with later cardiometabolic risk, although their individual contributions are difficult to disentangle. The clinically relevant phenotype is therefore not simply birth weight, but the trajectory of growth from fetal life through infancy and childhood. Rapid postnatal weight gain, particularly following fetal growth restriction or prematurity, may reflect a mismatch between developmental adaptation and the postnatal nutritional environment. Such trajectories have been associated with later adiposity, insulin resistance, hypertension, and cardiometabolic disease. This concept is consistent with the broader DOHaD framework, in which early developmental adaptations may become maladaptive when the subsequent environment differs substantially from that anticipated during fetal development. Childhood adiposity may consequently represent a visible clinical manifestation of a much longer metabolic trajectory rather than an isolated pediatric condition. At the same time, this relationship should not be interpreted deterministically. Genetic susceptibility, family environment, diet, physical activity, socioeconomic conditions, sleep, and subsequent exposures all contribute to the evolution of metabolic phenotypes. The value of an early-life perspective is therefore to identify a period in which metabolic trajectories may be particularly plastic, rather than to attribute later obesity or cardiometabolic disease to a single prenatal or infant exposure [75].

## 8. Preventive Strategies to Promote Lifelong Health

If early-life phenotypes can be interpreted as manifestations of altered biological trajectories, an important question is whether these trajectories remain modifiable. Prevention during the first 1000 days may support developmental and biological resilience through adequate nutrition, appropriate growth, maternal health, and protection from adverse environmental and psychosocial exposures. However, biological plausibility should not be equated with demonstrated long-term clinical efficacy. Although early-life interventions are increasingly supported by mechanistic and epidemiological evidence, relatively few studies have directly demonstrated that modifying an exposure during the first 1000 days prevents disease decades later. A recent systematic review of nutritional interventions in the first 1000 days found some evidence of benefit for cardiometabolic health and dietary behavior, but no effect on mental health and no randomized trial data on respiratory outcomes; most studies also had relatively short follow-up [76]. Preventive implications should therefore distinguish established maternal and child health benefits from the still uncertain potential to modify biological aging or lifelong health trajectories. Prevention begins during pregnancy, when maternal health and nutrition contribute to the intrauterine environment [77]. Maternal diet can influence the placental biochemical environment and nutrient supply to the fetus [78], while maternal nutritional status has been associated with offspring neurodevelopment; for example, higher prenatal vitamin B12 intake has been linked to better language and cognitive abilities in children [79]. Established preventive priorities also include appropriate gestational weight gain, prevention and management of obesity and gestational diabetes, avoidance of tobacco exposure, management of maternal disease, and reduction in preventable environmental exposures. Maternal metabolic disease, inflammation, oxidative stress, and environmental pollutants may converge on pathways involved in developmental programming [11,14,15]. Prenatal exposure to air pollution, tobacco smoke, and other pollutants has also been associated with alterations in placental, mitochondrial, inflammatory, and epigenetic pathways [13,14]. These findings provide biological plausibility for preventive approaches, although they do not demonstrate that modifying these pathways will prevent disease decades later. Maternal nutrition also influences the infant nutritional environment during breastfeeding. Maternal diet can affect breast-milk composition and thereby contribute to the infant’s early exposure to nutrients and bioactive compounds potentially relevant to development and long-term health [80,81]. Breastfeeding should, however, be considered within a broader nutritional and social context rather than as an isolated determinant of lifelong health, as associations with long-term outcomes are influenced by maternal, infant, and socioeconomic factors [82]. Despite its established role in infant nutrition and development, its potential influence on long-term biological aging remains uncertain. During infancy, growth monitoring represents another potentially important preventive strategy. Rapid early weight gain has been associated with increased risk of later overweight and obesity [76,77,83], although these associations do not imply that restricting physiological growth would improve long-term outcomes. Both inadequate growth and excessive postnatal weight gain may indicate altered developmental adaptation; therefore, longitudinal assessment of growth trajectories is preferable to reliance on isolated measurements of birth weight or body size. With complementary feeding, dietary variety, nutritional adequacy, and feeding practices become increasingly relevant. Early exposure to fruits and vegetables has been associated with greater subsequent liking of these foods, while food diversity and timing of introduction have been associated with allergic outcomes [84,85,86,87]. The introduction of complementary foods therefore represents not only a source of nutrients but also an important period of metabolic, microbial, and immunological adaptation. Current recommendations support complementary feeding from around six months, with progressive dietary diversity, nutritional adequacy, food safety, and responsiveness to the child’s hunger and satiety signals [88]. These practices have established nutritional and developmental relevance, whereas their potential effects on lifelong biological resilience remain less directly demonstrated. Adequate macro- and micronutrient intake is essential for neurological and somatic development during the first 1000 days [89,90]. Nutrients involved in one-carbon metabolism, including folate, vitamin B12, choline, and betaine, participate in pathways relevant to epigenetic regulation [91,92]. However, mechanistic involvement does not establish that supplementation with individual methyl donors can durably modify epigenetic programming or prevent long-term disease, and translation of associations between altered methylation and metabolic or inflammatory disorders into specific preventive interventions remains uncertain [93]. A similar distinction applies to antioxidants. Oxidative stress is implicated in pathways involving inflammation, mitochondrial dysfunction, and cellular aging, but this mechanistic rationale does not establish high-dose antioxidant supplementation as a strategy for lifelong disease prevention. Clinical and translational evidence has not demonstrated consistent preventive benefits from indiscriminate supplementation with individual antioxidant vitamins [94,95,96,97]. Current evidence therefore supports adequate nutritional status and overall dietary quality rather than supplementation with individual components of redox homeostasis. Dietary patterns rich in fruits and vegetables have been associated more reliably with favorable health outcomes than isolated antioxidant supplementation [98]. Prevention during the first 1000 days also extends beyond individual behavior. Socioeconomic conditions influence maternal and infant nutrition, psychosocial stress, environmental exposures, and access to healthcare. A systematic review of income-support interventions reported small improvements in birth weight and selected maternal and child mental health outcomes [99], suggesting that interventions addressing social determinants can modify some early-life exposures and outcomes. Table 1 summarizes illustrative early-life phenotypes, potential modifiable targets, and corresponding preventive opportunities during the first 1000 days.

These findings support integrating nutritional and clinical care with public-health policies and social measures, while evidence for effects extending into adult healthspan remains limited. Overall dietary quality may therefore be more clinically relevant than focusing on isolated nutrients. A diverse and nutritionally adequate dietary pattern, with limited intake of foods rich in free sugars and other energy-dense, nutrient-poor products, represents an important modifiable component of chronic disease prevention [100,101]. Taken together, prevention during the first 1000 days should be viewed as a multidimensional strategy encompassing maternal health, adequate nutrition, breastfeeding, complementary feeding, appropriate growth, protection from avoidable environmental exposures, and favorable social conditions. Many of these measures have established benefits for maternal and child health, whereas evidence that they specifically modify biological aging or prevent disease decades later remains limited.

## 9. Strengths and Limitations

Strengths: This review adopts a multidisciplinary perspective, bringing together developmental biology, clinical pediatrics, nutrition, environmental health, and geroscience within a single framework. It integrates experimental evidence on epigenetic regulation, mitochondrial function, redox balance, inflammation, and telomere biology with human observational and interventional data, indicates the type of evidence supporting each proposed link, and incorporates literature published up to June 2026. Finally, it attempts to bridge pediatrics and geroscience by proposing clinically observable pediatric phenotypes as potential early read-outs of processes relevant to biological aging.

Limitations: This is a narrative rather than a systematic review: the search was non-systematic, no protocol was registered, and no structured appraisal of methodological quality or risk of bias was performed. Study selection therefore reflects the authors’ judgment and is subject to selection bias, while reliance on published literature may introduce publication bias. The included studies are heterogeneous in design, population, exposure window, follow-up, and outcome definition. The biomarkers discussed are also heterogeneous, differing in tissue source, standardization, and measurement error, and are not interchangeable. A substantial proportion of the mechanistic evidence derives from animal and in vitro models and cannot be directly extrapolated to humans, whereas most human evidence is observational and cannot exclude residual confounding or reverse causation. The relationships described here should therefore be interpreted as associations and biologically plausible hypotheses rather than as demonstrated causal pathways. Very long-term longitudinal data remain scarce, and the translational chain presented here represents a conceptual model assembled from different study populations. Finally, the four sentinel phenotypes were selected as illustrative examples rather than through a formal procedure.

## 10. Conclusions

The first 1000 days represent a period of exceptional biological plasticity during which nutritional, metabolic, inflammatory, psychosocial, microbial, and environmental signals interact with rapidly developing physiological systems. Within the Developmental Origins of Health and Disease (DOHaD) framework, these exposures may influence developmental trajectories through interconnected mechanisms involving epigenetic regulation, oxidative stress, mitochondrial function, chronic inflammation, and telomere biology [2,3,4,8,9,10,11,12,13,14,15,25,26,27,30,31,34,35,36,37]. Increasing evidence also suggests that adverse early-life circumstances are associated with measures of biological aging across multiple organ systems, although the strength and causality of these associations remain heterogeneous. Importantly, early-life programming should not be interpreted as a deterministic process. The biological effects of an exposure depend on its timing, intensity, duration, tissue specificity, and interaction with subsequent environmental conditions. Their clinical consequences may become apparent during childhood as sentinel phenotypes through which altered biological trajectories become clinically visible. Allergic and atopic diseases, respiratory vulnerability, renal–cardiovascular risk, and altered metabolic growth provide illustrative examples, although their presence should not be interpreted as a direct prediction of future adult disease. The same distinction applies to prevention. The first 1000 days provide an important opportunity to optimize maternal health, nutrition, growth, environmental conditions, and access to appropriate healthcare. These measures have established benefits for maternal and child health, whereas evidence that they specifically modify biological aging or prevent chronic disease decades later remains limited. Prevention should therefore focus on creating a favorable developmental environment rather than attempting to manipulate individual molecular pathways in isolation. Within this framework, different health trajectories may emerge depending on the organ system involved, the nature and timing of the exposure, and the developmental context. This perspective supports a life-course model in which developmental adaptation, biological resilience, and subsequent environmental exposures continuously interact (Figure 1).

**Figure 1:**
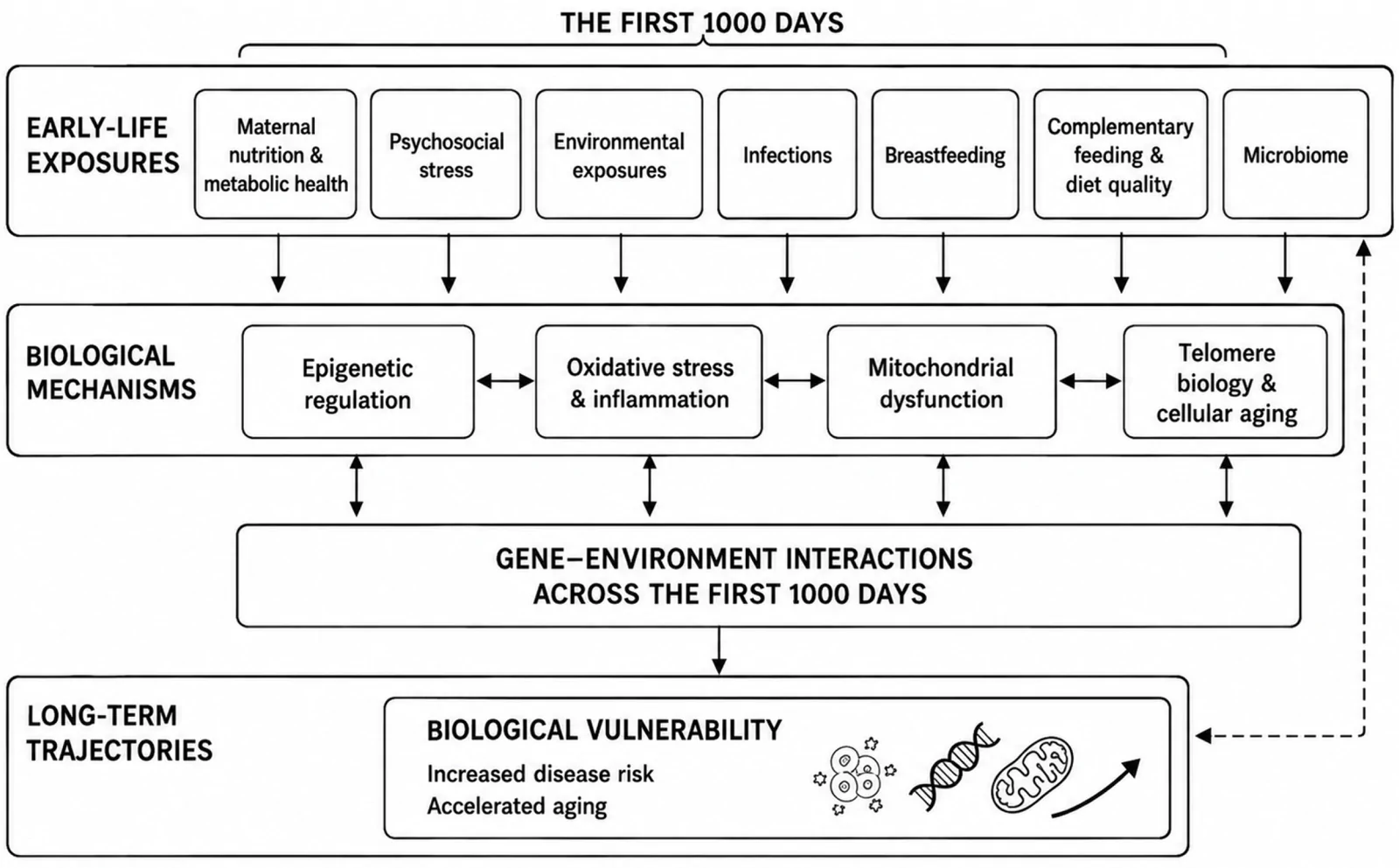
Conceptual model linking early-life exposures, biological programming, and long-term health trajectories. Arrows indicate proposed or hypothesized relationships rather than established causal pathways in humans.

Ultimately, the first 1000 days should be regarded as a framework for understanding the early-life foundations of healthy aging rather than as a prediction of later-life health. Biological processes relevant to healthy aging may begin early in life, while subsequent trajectories remain modifiable. The pediatric period may therefore represent one of the earliest clinically accessible windows in which sentinel phenotypes can be recognized and biological resilience potentially supported. Further longitudinal studies integrating molecular biomarkers with clinically meaningful outcomes are needed to determine how strongly early-life biological signatures predict aging trajectories and whether interventions during this developmental window can meaningfully alter healthspan and later-life disease risk.

## Figures and Tables

**Table 1:** Illustrative examples of early-life phenotypes and opportunities for prevention during the first 1000 days. Details of the main supporting studies are reported in the Supplementary Materials (Table S1).

Early-Life Phenotype/Trajectory	Potential Early-Life Driver	Potentially Modifiable Target	Example of Plausible Intervention	Level/Type of Evidence	References
Allergic and atopic trajectory (atopic dermatitis)	Early-life dietary exposures and environmental factors	Immune tolerance, feeding practices	Promoting dietary diversity during complementary feeding	Human observational evidence	[86,87]
Respiratory vulnerability (recurrent wheezing/asthma)	Maternal smoking/air pollution, prematurity, early respiratory infections, altered immune development	Environmental exposure, respiratory infection prevention, airway inflammation	Reduction in tobacco smoke and relevant air-pollution exposure	Human observational evidence	[73]
Early renal–cardiovascular risk	Prematurity, fetal growth restriction, maternal hypertension/diabetes, rapid postnatal growth	Blood pressure, growth, renal function, cardiovascular risk factors	Avoidance of excessive postnatal weight gain; targeted blood pressure and renal surveillance in at-risk infants	Human observational evidence; consensus guideline	[54,68,74]
Altered metabolic growth (excessive weight gain/early adiposity)	Maternal obesity and metabolic disease, excessive gestational weight gain, rapid infant weight gain, inappropriate feeding, diet quality	Growth trajectory, adiposity, insulin sensitivity, feeding practices, parental behaviors	Optimization of maternal metabolic health; breastfeeding support; responsive feeding; appropriate complementary feeding	Human observational evidence; RCT evidence for feeding interventions; consensus guideline	[63,76,83,88]

## Data Availability

No new data were created or analyzed in this study. Data sharing is not applicable to this article.

## Supplementary Materials

The following supporting information can be downloaded at https://www.mdpi.com/article/10.3390/children13101302/s1, Table S1: Summary of key studies included in the review.

## Abbreviations

The following abbreviations are used in this manuscript:

AD	atopic dermatitis
cGAS	cyclic GMP-AMP synthase
DAMP	damage-associated molecular pattern
DOHaD	Developmental Origins of Health and Disease
HMO	human milk oligosaccharide
HPA	hypothalamic–pituitary–adrenal
IGF2	insulin-like growth factor 2
mtDNA	mitochondrial DNA
NLRP3	NOD-, LRR-, and pyrin domain-containing protein 3
ROS	reactive oxygen species
RSV	respiratory syncytial virus
SGA	small for gestational age
STING	stimulator of interferon genes
