# Supplementary material for: From the First 1000 Days to the First 100 Years: Early-Life Biological Programming, Sentinel Phenotypes, and Healthy Aging
Source: Children (Basel). 2026 Sep 25;13(10):1302. doi: 10.3390/children13101302 (PMC13703749; doi:10.3390/children13101302)
Supplement: Supplementary file 1 [file children-13-01302-s001.zip › children-4567297-supplementary.pdf]

Table S1: Summary of key studies included in the review.

Study	Study type	Population	Contribution to our review	Reference
**Shrestha D, et al. 2020**	Epigenome-wide association study	Human	Maternal prepregnancy BMI and gestational weight gain are associated with differences in placental DNA methylation, supporting the sensitivity of the placental epigenome to maternal metabolic conditions; it does not establish that these methylation differences cause later metabolic disease or biological aging.	[10]
**Lim AI, et al. 2021**	Experimental study	Animal (mice)	Maternal infection can induce IL-6-dependent epigenetic changes in fetal intestinal epithelial stem cells, resulting in persistent changes in offspring immunity; it illustrates a developmental trade-off between enhanced antimicrobial protection and increased inflammatory susceptibility, but its relevance to long-term human aging remains to be established.	[15]
**Coker MO, et al. 2021**	Prospective longitudinal cohort study	Human	Infant feeding modifies the longitudinal impact of birth mode on the development of the infant gut microbiota during the first year of life, supporting the role of interacting early-life exposures in shaping microbiome development.	[19]
**Jiménez-Loygorri JI, et al. 2024**	Experimental mechanistic study	Cellular and mouse models	Mitophagy restrains cytosolic mtDNA-dependent cGAS–STING inflammatory signaling during aging, providing a mechanistic link between mitochondrial quality control and inflammaging; it does not establish that early-life alterations in mitophagy determine subsequent human aging trajectories.	[31]
**Gulen MF, et al. 2023**	Experimental mechanistic study	Mice and human cells/tissues	cGAS-–STING signaling contributes to age-associated inflammation and functional decline, with STING inhibition attenuating inflammatory and neurodegenerative phenotypes in aged mice; it does not demonstrate that this pathway is programmed by early-life exposures in humans.	[35]
**Victorelli S, et al. 2023**	Experimental mechanistic study	Cellular and mouse models	Mitochondrial DNA release during cellular senescence activates cGAS–STING signaling and promotes the senescence-associated secretory phenotype (SASP), linking mitochondrial dysfunction to inflammatory aging mechanisms; it does not establish a longitudinal connection between early-life mitochondrial changes and human aging.	[38]
**Heijmans BT, et al. 2008**	Retrospective cohort study	Human	A narrow prenatal window may leave a durable molecular signature; it does not establish that these differences caused later disease.	[37]
**Treviño LS, et al. 2020**	Experimental study	Animal (mice)	Early-life chemical exposure reprogrammed hepatic histone marks and accelerated<br>acquisition of an adult epigenomic signature; metabolic dysfunction emerged only after<br>a later dietary challenge. Gives experimental support to the mismatch model, in which<br>early programming is unmasked by a subsequent exposure	[53]
**Musci RJ, et al. 2023**	Systematic review of observational studies	Human	By reporting significant associations between early-life exposures, epigenetic age, and<br>health outcomes, connects early-life exposures to measures of biological aging already<br>within the pediatric age range, supporting the link between the first 1,000 days and<br>biological aging research.	[55]

Study	Study type	Population	Contribution to our review	Reference
**Broer L, et al. 2013**	Meta-analysis of observational studies	Human	It supports a substantial heritable contribution to telomere length and suggests that telomere length is influenced by factors acting before or around birth.	[58]
**Ye Q, et al. 2023**	Systematic review and meta-analysis of observational studies	Human	It anchors telomere length as an age-related marker at the population level (inverse association between telomere length and chronological age), while quantifying the heterogeneity that limits its interpretation in individuals.	[60]
**Moshfeghinia R, et al. 2023**	Systematic review and meta-analysis of observational studies	Human	An inverse association was found between maternal prenatal stress and newborn telomere length, suggesting that a prenatal psychosocial exposure may be associated with telomere biology already at birth.	[65]
**Hsu CN, et al. 2021**	Narrative review	Human and animal (predominantly rats)	It supports the first 1,000 days as a critical window for kidney development and renal programming, linking maternal and early-life exposures to later kidney health and highlighting potential preventive strategies.	[68]
**Hartert T, et al. 2025**	Narrative review	Human	Birth-cohort evidence linking prenatal and early-life exposures with later respiratory health. Supports critical periods of developmental plasticity and susceptibility for asthma and chronic respiratory phenotypes.	[73]
**Wu AJ, et al. 2023**	Narrative review	Human	Maternal and infant nutritional exposures were associated with subsequent adiposity and obesity risk. Suggests that early-life nutrition may influence metabolic trajectories relevant to childhood obesity	[75]
**Xu A, et al. 2025**	Systematic review of randomized controlled trials	Human	It reports some beneficial effects of nutrition interventions during the first 1000 days on cardiometabolic health and dietary behaviour, although findings were heterogeneous and long-term follow-up was limited. Supports the potential role of early nutritional interventions in shaping later health outcomes.	[76]
**Blaauwendraad SM, et al. 2026**	Systematic review of observational and intervention studies	Human	It identifies multiple maternal, perinatal, and infant risk factors associated with childhood obesity. Supports an association between first-1,000-days exposures and subsequent metabolic development, while highlighting heterogeneity and moderate evidence strength.	[77]
**Roduit C, et al. 2014**	Prospective observational study	Human	An inverse association was found between complementary food diversity during the first year of life and subsequent asthma, food allergy, and food sensitization. Suggests that early-life dietary diversity may be relevant to later allergic outcomes.	[86]
**Boccia D, et al. 2023**	Systematic review of experimental and quasi-experimental studies	Human	Income-support interventions were associated with small positive effects on some early-life health outcomes, particularly birth weight, supporting socioeconomic conditions as potentially modifiable determinants of early-life health.	[99]
